# The clinical profiles of female patients with Fabry disease in Latin America: A Fabry Registry analysis of natural history data from 169 patients based on enzyme replacement therapy status

**DOI:** 10.1002/jmd2.12071

**Published:** 2019-08-05

**Authors:** Ana M. Martins, Gustavo Cabrera, Fernando Molt, Fernando Suárez‐Obando, Régulo A. Valdés, Carmen Varas, Meng Yang, Juan M. Politei

**Affiliations:** ^1^ Reference Center for Inborn Errors of Metabolism Federal University of São Paulo São Paulo SP Brazil; ^2^ Centro Médico Santa María de la Salud Buenos Aires Argentina; ^3^ Departamento de Clínicas, Facultad de Medicina Universidad Católica del Norte Coquimbo Chile; ^4^ Fabry Disease Multidisciplinary Team Hospital San Pablo de Coquimbo Coquimbo Chile; ^5^ Instituto de Genética Humana, Facultad de Medicina, Hospital Universitario San Ignacio Pontificia Universidad Javeriana Bogota Colombia; ^6^ National Dialysis Coordinator of Social Security of Panama Panama City Panama; ^7^ Sanofi Genzyme Cambridge Massachusetts; ^8^ Department of Neurology Fundación Para el Estudio de Enfermedades Neurometabólicas (FESEN) Buenos Aires Argentina

**Keywords:** diagnostic delay, enzyme replacement therapy, Fabry disease, females, Latin America, registry

## Abstract

**Background:**

Fabry disease is an X‐linked lysosomal storage disorder with heterogeneous clinical expression in female patients ranging from asymptomatic to severe clinical presentations as in classic males. We assessed clinical profiles and compared natural history data of female patients eventually initiated on enzyme replacement therapy (“ERT‐recipients”) with those remaining untreated (“ERT‐naïve”).

**Methods:**

We analyzed Fabry Registry data from 93 ERT‐recipients, collected prior to ERT initiation, and 76 ERT‐naïve females with classic or unclassified phenotypes from four Latin American countries and evaluated Fabry symptoms, interventricular septum thickness, left ventricular posterior wall thickness, estimated glomerular filtration rate, and severe clinical events.

**Results:**

For 169 patients with available data, median age of first Fabry symptom manifestation was 12.7 years with peripheral pain as predominant first symptom, and diagnostic delay of 10.3 years from the first reported symptom. Female patients had high symptomatic burden during natural history follow‐up, with 83% reporting peripheral pain, 69%‐79% cold/heat intolerance or abnormal sweating, and 32% gastrointestinal symptoms. ERT‐recipients reported similar age at first symptom as ERT‐naïve patients but they were older at diagnosis (median 39.2 vs 24.4 years, *P* < .01) and last follow‐up (median 43.4 vs 28.2 years, *P* < .01). Reported Fabry symptom frequencies and abnormal echocardiography findings were higher in ERT‐recipients. Functional renal assessments were normal and similar.

**Conclusions:**

Female patients from Latin America have notable diagnostic delays and high symptomatic burden. ERT was prescribed late in females with advanced age at diagnosis and advanced disease. There remained many female patients who had been diagnosed at younger age, had substantial Fabry manifestations, but did not receive disease‐specific treatment.

## 1 INTRODUCTION

Fabry disease (OMIM 301500) is a progressive X‐linked lysosomal storage disorder. Pathogenic variants of the *GLA* gene encoding α‐galactosidase (α‐Gal) result in reduced or abolished enzyme activity and allow accumulation of substrates (eg, globotriaosylceramide [GL‐3] and deacylated GL‐3 [lyso‐GL‐3]) in plasma and a variety of cell types.^1^ Males with *GLA* variants associated with the classic phenotype are at risk of developing severe end‐organ complications involving the kidneys, heart, and brain.^2^ Female patients with Fabry disease are no longer believed to be mere “carriers” of the disease since they may experience, for example, peripheral pain, gastrointestinal (GI) symptoms, impaired sweating, angiokeratomas, and cornea verticillata, impairing quality of life.^3^, ^4^ The phenotypic spectrum of female patients is heterogenous depending, in part, on the type of mutation, the level of residual α‐Gal activity, and the pattern of (skewed) X‐chromosome inactivation (lyonization) in various organs.^5^, ^6^ Therefore, some female patients can be asymptomatic while others have severe progression with vital organ complications (similar to classically affected males) reducing their lifespan.^2^, ^4^, ^7^ Available enzyme replacement therapies (ERTs) include agalsidase beta (1 mg/kg body weight every 2 weeks [EOW]) and agalsidase alfa (0.2 mg/kg EOW). Chaperone therapy is restricted to patients with amenable mutations and is only approved in certain countries.^8^ Regular monitoring is necessary to facilitate therapeutic decisions and published guidelines provide clear recommendations for initiation of disease‐specific treatment in female patients with Fabry disease.^8^, ^9^, ^10^ The aim of our study was to assess the demographic and clinical profiles of female Fabry patients enrolled in the Fabry Registry in major Latin American countries and to compare natural history data from female patients who were eventually initiated on ERT with data from females who remained untreated.

## 2 METHODS

### 2.1 Fabry Registry

The Fabry Registry is a global, longitudinal, observational program designed to track the natural history and treatment outcomes of patients with Fabry disease. It was initiated in 2001 in an effort to help practitioners involved in the diagnosis and/or treatment of Fabry disease to increase their understanding of the disease, facilitate clinical management, and develop Fabry disease treatment monitoring guidelines. Patient and investigator participations are voluntary. Recommended schedules of clinical assessments are available (www.fabrydisease.org/images/ReferencePDFs/fabry-registry-schedule-of-assessments.pdf), but treating physicians determine the actual frequency of assessments according to a patient's individualized need for medical care and routine follow‐up. The Fabry Registry is registered at www.ClinicalTrials.gov (NCT00196742) and is sponsored by Sanofi Genzyme.

### 2.2 Patients

Females with Fabry disease of all ages from Brazil, Chile, Argentina, and Colombia who had been enrolled in the Fabry Registry and had the natural history data of interest (ie, collected during the period when patients were not receiving ERT) were included in the analysis. Natural history data from females who were subsequently initiated on ERT (“ERT‐recipients”) were compared with those from females who were not treated with ERT (“ERT‐naïve”).

The analysis population was restricted to females with *GLA* variants categorized in the fabry-database.org *GLA* variant database (http://fabry-database.org/mutants/) as being associated with “classic” Fabry disease or variants reported to the Fabry Registry but not entered or classified in this database. We excluded patients with reported *GLA* variants classified as later‐onset phenotypes and variants previously reported to be of uncertain significance, as well as patients whose genotype had not been reported to the Fabry Registry.

### 2.3 Demographic and clinical assessments

We analyzed demographic and clinical data on Fabry disease presentation and manifestations collected during the natural history period. Echocardiographic assessments included interventricular septum thickness (IVST) and left ventricular posterior wall thickness (LVPWT). Assessments of renal function were estimated glomerular filtration rate (eGFR; Bedside Schwartz equation if <18 years of age or Chronic Kidney Disease Epidemiology Collaboration [CKD‐EPI] creatinine equation if ≥18 years of age), urine albumin‐to‐creatinine ratio (UACR), and urine protein‐to‐creatinine ratio (UPCR). Severe clinical events were defined as: (a) cardiovascular events: significant cardiac procedure (ie, balloon pump, angioplasty with stent, bypass, implantation of a cardioverter/defibrillator or pacemaker), arrhythmia (atrial fibrillation, ventricular tachycardia), angina pectoris, myocardial infarction, congestive heart failure, cardiac syncope; (b) renal events: chronic dialysis, kidney transplantation; and (c) cerebrovascular events: stroke, transient ischemic attacks (TIAs).

### 2.4 Statistical analyses

Descriptive statistics were calculated for demographic and clinical data. Numbers of patients who ever reported clinical assessments or events were obtained. The last assessment along with the corresponding age parameters during the natural history period was estimated and the last assessment was defined as the last value of any given parameter within a 2‐year window before the end of natural history follow‐up in ERT‐naïve patients, or a 2‐year window before the treatment initiation in ERT‐recipients. We also reported age at the first clinical assessment or age at the first event. In addition, IVST, LVPWT and eGFR assessments were analyzed cross‐sectionally by grouped age at assessment: 18 to <30 years, 30 to <40 years, 40 to <50 years, 50 to <60 years, and ≥60 years.

Continuous variables were analyzed using the Wilcoxon signed rank test and categorical variables using a chi‐squared or Fisher's exact test between ERT‐recipients and ERT‐naïve patients. All statistical tests were two‐sided and *P* < 0.05 was considered to represent statistical significance. Statistical analyses were performed using SAS statistical software V.9.2 (SAS Institute, Cary, NC).

## 3 RESULTS

### 3.1 Patient demographics

Patient demographics are summarized in Table 1. The Fabry Registry observation period extended through March 2, 2018. Natural history data from 169 female patients were analyzed, including 93 (55.0%) ERT‐recipients and 76 (45.0%) ERT‐naïve patients (Brazil, n = 62 and 42, respectively; Chile, n = 15 and 16; Argentina, n = 14 and 15; Colombia, n = 2 and 3). Genotypes were available for all patients. *GLA* variants associated with a classic phenotype were reported for 46.7% of the female patients and 53.3% had reported *GLA* variants either not listed in the fabry-database.org database or listed without a phenotype classification. The phenotype percentages for ERT‐recipients and ERT‐naïve patients were similar.

**Table 1:** Patient demographics

	ERT‐recipients (n = 93)	ERT‐naïve (n = 76)	*P* value	Overall (n = 169)
Country, n (%)				
Brazil	62 (66.7)	42 (55.3)		104 (61.5)
Chile	15 (16.1)	16 (21.1)		31 (18.3)
Argentina	14 (15.1)	15 (19.7)		29 (17.2)
Colombia	2 (2.2)	3 (3.9)		5 (3.0)
Ethnicity, n (%)				
Hispanic or Latino	21 (22.6)	33 (43.4)		54 (32.0)
Not Hispanic or Latino	0	0		0
Not available	72 (77.4)	43 (56.6)		115 (68.0)
FD phenotype, n (%)				
Classic	42 (45.2)	37 (48.7)		79 (46.7)
Unclassified^a^	51 (54.8)	39 (51.3)		90 (53.3)
FD family history, n (%)	85 (91.4)	61 (80.3)	.04	146 (86.4)
Age at first FD manifestation				
N (%)	56 (60.2)	38 (50.0)		94 (55.6)
Mean (SD)	18.5 (15.2)	15.2 (10.9)	.58	17.1 (13.7)
Median, range	12.8, 0.6‐67.9	12.7, 0‐48.9		12.7, 0‐67.9
Age at FD diagnosis				
N (%)	92 (98.9)	74 (97.4)		166 (98.2)
Mean (SD)	37.1 (16.6)	26.2 (17.2)	<.01	32.2 (17.7)
Median, range	39.2, 1.1‐72.0	24.4, 0.4‐71.8		29.9, 0.4‐72.0
Time from first FD manifestation to diagnosis				
N (%)	55 (59.1)	37 (48.7)		92 (54.4)
Mean (SD)	17.1 (17.2)	11.7 (11.7)	.34	14.9 (15.4)
Median, range	13.1, −5.9 to 62.0	7.8, −0.6 to 49.1		10.3, −5.9 to 62.0
Time from first to last NH assessment^b^				
N (%)	88 (94.6)	69 (90.8)		157 (92.9)
Mean (SD)	15.7 (16.9)	11.6 (12.8)	.36	13.9 (15.3)
Median, range	7.5, 0‐63.2	7.2, 0‐64.4		7.5, 0–64.4
Age at last NH assessment^b^				
N (%)	88 (94.6)	69 (90.8)		157 (92.9)
Mean (SD)	40.9 (15.8)	30.7 (17.5)	<.01	36.4 (17.3)
Median, range	43.4, 12.9‐73.7	28.2, 1.1‐76.8		34.4, 1.1‐76.8
Age at ERT initiation				
N (%)	93 (100)	–	–	–
Mean (SD)	40.3 (16.1)	–		–
Median, range	43.2, 3.8‐73.7	–		–

*Note*: All ages and durations in years. Percentages are based on the total number of patients in each group.

Abbreviations: ERT, enzyme replacement therapy; FD, Fabry disease; NH, natural history.

a *GLA* variant entered into the Fabry Registry but not listed or classified in the fabry‐database.org database.

b Last NH assessment = most recent value within a 2‐year window before start of ERT (“ERT‐recipients”) or before the last follow‐up date (“ERT‐naïve”).

A total of 86.4% of the patients had a family history of Fabry disease and the ERT‐recipients group had a significantly higher family history compared with the ERT‐naïve group (91.4% vs 80.3%, *P* = 0.04). Data on the type of first (any) manifestation of Fabry disease and age at onset were available for approximately half of the patients (Table S1). The first manifestation occurred at a median age of 12.7 years, which was similar for both groups (12.8 vs 12.7 years, *P* = 0.58). Peripheral pain was predominantly reported at onset (64.9% of the total population, 66.1% of ERT‐recipients, 63.2% of ERT‐naïve patients). The median age of disease diagnosis was 29.9 years, with ERT‐recipients being significantly older at diagnosis (39.2 vs 24.4 years, *P* < 0.01). For 92 patients with both age parameters available, the median time from the first Fabry disease manifestation to diagnosis was 10.3 years and, although longer for ERT‐recipients, statistically comparable for the two groups (13.1 vs 7.8 years, *P* = 0.34) (Table 1). The median time from the first to last natural history record was 7.5 years and did not significantly differ between groups (7.5 vs 7.2 years, *P* = 0.36), but ERT‐recipients had a significantly older age at last natural history follow‐up (median 43.4 vs 28.2 years, *P* < 0.01). In ERT‐recipients, treatment was initiated at a median age of 43.2 years.

### 3.2 Fabry symptom reports during follow‐up

Frequencies of Fabry symptoms reported during natural history follow‐up and ages at onset are presented in Table 2. The percentage of patients who had ever reported peripheral pain was higher for ERT‐recipients than for ERT‐naïve patients (96.5% vs 70.5%, overall 83.1%) and similar patterns were found for abnormal sweating (90.9% vs 69.6%, overall 79.0%), and heat (93.5% vs 65.5%, overall 78.2%), and cold intolerance (90.2% vs 54.4%, overall 69.4%). Reports of having ever experienced acute pain crisis (35.8% vs 24.6%, overall 29.6%), abdominal pain (42.9% vs 23.6%, overall 32.0%), diarrhea (41.8% vs 23.6%, overall 31.5%), and angiokeratoma (32.7% vs 25.4%, overall 28.5%) were less frequent but also occurred more often in ERT‐recipients. The median ages of the patients at onset of the various Fabry symptoms were numerically higher for ERT‐recipients; nonetheless, the age difference was statistically significant only for abdominal pain (28.9 vs 15.4 years, *P* < 0.05) and abnormal sweating (37.2 vs 23.3 years, *P* = 0.04). For all types of Fabry symptoms, the frequency of reports at the last natural history assessment were lower as compared with frequencies observed during the entire follow‐up period (ever reported). The percentages of patients with available data were substantially lower for ERT‐recipients for all Fabry symptoms.

**Table 2:** Fabry disease symptoms reported during natural history follow‐up in the Fabry Registry

FD symptoms	Statistics	ERT‐recipients (n = 93)	ERT‐naïve (n = 76)	*P* value	Overall (n = 169)
Abdominal pain	NH data, n (%)	56 (60.2)	72 (94.7)		128 (75.7)
	Ever present, n (%)	24 (42.9)	17 (23.6)		41 (32.0)
	Age at first report				
	Mean (SD)	30.4 (17.2)	18.8 (14.7)	<.05	25.6 (17.0)
	Median, range	28.9, 0‐56.6	15.4, 0.9‐53.6		22.8, 0–56.6
	Last NH assessment data,^a^ n (%)	48 (51.6)	63 (82.9)		111 (65.7)
	Present at last NH assessment,^a^ n (%)	15 (31.2)	10 (15.9)		25 (22.5)
Diarrhea	NH data, n (%)	55 (59.1)	72 (94.7)		127 (75.1)
	Ever present, n (%)	23 (41.8)	17 (23.6)		40 (31.5)
	Age at first report				
	Mean (SD)	29.8 (16.2)	20.6 (16.7)	.10	25.9 (16.9)
	Median, range	25.7, 0‐58.9	18.7, 0‐53.6		22.9, 0‐58.9
	Last NH assessment data,^a^ n (%)	46 (49.5)	63 (82.9)		109 (64.5)
	Present at last NH assessment,^a^ n (%)	13 (28.3)	11 (17.5)		24 (22.0)
Peripheral pain	NH data, n (%)	57 (61.3)	61 (80.3)		118 (69.8)
	Ever present, n (%)	55 (96.5)	43 (70.5)		98 (83.1)
	Age at first report				
	Mean (SD)	32.2 (17.1)	27.1 (17.9)	.08	29.9 (17.5)
	Median, range	26.0, 0‐62.5	23.3, 1.4‐74.2		24.6, 0‐74.2
	Last NH assessment data,^a^ n (%)	38 (40.9)	54 (71.1)		92 (54.4)
	Present at last NH assessment,^a^ n (%)	34 (89.5)	34 (63.0)		68 (73.9)
Acute pain crisis	NH data, n (%)	56 (60.2)	69 (90.8)		125 (74.0)
	Ever present, n (%)	20 (35.8)	17 (24.6)		37 (29.6)
	Age at first report				
	Mean (SD)	31.3 (14.1)	25.4 (14.4)	.26	28.6 (14.3)
	Median, range	31.4, 8.7‐49.9	24.9, 2.7‐56.5		27.9, 2.7–56.5
	Last NH assessment data,^a^ n (%)	47 (50.5)	61 (80.3)		108 (63.9)
	Present at last NH assessment,^a^ n (%)	8 (17.0)	8 (13.1)		16 (14.8)
Sweating abnormal	NH data, n (%)	44 (47.3)	56 (73.7)		100 (59.2)
	Ever present, n (%)	40 (90.9)	39 (69.6)		79 (79.0)
	Age at first report				
	Mean (SD)	35.0 (17.5)	26.5 (17.1)	.04	30.8 (17.7)
	Median, range	37.2, 0‐62.5	23.3, 0‐68.3		25.8, 0‐68.3
	Last NH assessment data,^a^ n (%)	32 (34.4)	51 (67.1)		83 (49.1)
	Present at last NH assessment,^a^ n (%)	25 (78.1)	30 (58.5)		55 (66.3)
Heat intolerance	NH data, n (%)	46 (49.5)	55 (72.4)		101 (59.8)
	Ever present, n (%)	43 (93.5)	36 (65.5)		79 (78.2)
	Age at first report				
	Mean (SD)	31.1 (17.4)	26.5 (15.0)	.27	29.0 (16.4)
	Median, range	26.8, 0‐62.5	23.6, 0‐59.4		25.2, 0‐62.5
	Last NH assessment data,^a^ n (%)	33 (35.5)	47 (61.8)		80 (47.3)
	Present at last NH assessment,^a^ n (%)	28 (88.5)	23 (51.5)		51 (67.8)
Cold intolerance	NH data, n (%)	41 (44.1)	57 (75.0)		98 (58.0)
	Ever present, n (%)	37 (90.2)	31 (54.4)		68 (69.4)
	Age at first report				
	Mean (SD)	31.2 (17.3)	28.7 (16.3)	.62	30.1 (16.8)
	Median, range	25.8, 6.0‐62.5	23.7, 7.1‐72.5		25.0, 6.0‐72.5
	Last NH assessment data,^a^ n (%)	29 (31.2)	48 (63.2)		77 (45.6)
	Present at last NH assessment,^a^ n (%)	25 (86.2)	20 (41.7)		45 (58.4)
Angiokeratoma	NH data, n (%)	52 (55.9)	71 (93.4)		123 (72.8)
	Ever present, n (%)	17 (32.7)	18 (25.4)		35 (28.5)
	Age at first report				
	Mean (SD)	40.0 (13.0)	32.9 (14.5)	.21	36.4 (14.1)
	Median, range	43.2, 21.8‐66.1	30.3, 1.1‐59.4		36.3, 1.1‐66.1
	Last NH assessment data,^a^ n (%)	46 (49.5)	61 (80.3)		107 (63.3)
	Present at last NH assessment,^a^ n (%)	12 (26.1)	11 (18.0)		23 (21.5)

*Note*: All ages in years. Percentages are based on the total number of patients in each group.

Abbreviations: ERT, enzyme replacement therapy; FD, Fabry disease; NH, natural history.

a Last NH assessment = most recent value within a 2‐year window before start of ERT (“ERT‐recipients”) or before the last follow‐up date (“ERT‐naïve”).

### 3.3 Cardiac measures

Echocardiographic data of IVST and LVPWT were available for 54 ERT‐recipients (58.1%) and 44 ERT‐naïve females (57.9%) (Table 3). The first observation of an abnormal IVST result (≥10.0 mm^11^) was made at a similar median age in both groups (47.0 [n = 35, 64.8%] vs 46.0 years [n = 16, 36.4%]; *P* = 0.93). Last assessments of IVST were performed at an older median age in ERT‐recipients (46.1 [n = 40] vs 30.8 years [n = 26]). Median IVST values for both groups were similar (10.0 vs 9.0 mm), and the percentages of patients with a last assessment of ≥10.0 mm were 62.5% and 42.3%, respectively.

**Table 3:** Echocardiography assessments during natural history follow‐up in the Fabry Registry

	ERT‐recipients (n = 93)	ERT‐naïve (n = 76)	Overall (n = 169)
Patients with ever echo, n (%)^a^	54 (58.1)	44 (57.9)	98 (58.0)
IVST data, n (%)^a^	54 (58.1)	44 (57.9)	98 (58.0)
Age at first IVST ≥10.0 mm, years			
N (%)^b^	35 (64.8)	16 (36.4)	51 (52.0)
Mean (SD)	45.6 (11.3)^c^	46.7 (11.6)^c^	45.9 (11.3)
Median, range	47.0, 14.8‐65.4	46.0, 23.6‐71.8	46.0, 14.8‐71.8
Last IVST NH assessment,^d^ mm			
N (%)^b^	40 (74.1)	26 (59.1)	66 (67.3)
Mean (SD)	11.4 (4.1)	9.9 (3.3)	10.8 (3.9)
Median, range	10.0, 6.0‐23.0	9.0, 6.0‐20.0	10.0, 6.0‐23.0
IVST categories, n (%)^b^			
<10.0 mm	15 (37.5)	15 (57.7)	30 (45.5)
≥10.0 mm	25 (62.5)	11 (42.3)	36 (54.5)
Age at last IVST NH assessment,^d^ years			
N (%)^b^	40 (74.1)	26 (59.1)	66 (67.3)
Mean (SD)	41.5 (15.3)	36.4 (17.3)	39.5 (16.2)
Median, range	46.1, 14.7‐66.1	30.8, 5.7‐76.8	43.1, 5.7‐76.8
LVPWT data, n (%)^a^	54 (58.1)	44 (57.9)	98 (58.0)
Age at first LVPWT ≥10.0, years			
N (%)^b^	26 (48.1)	13 (29.5)	39 (39.8)
Mean (SD)	49.2 (8.3)^e^	47.3 (10.4)^e^	48.5 (8.9)
Median, range	49.5, 24.2‐65.4	46.0, 31.7‐71.8	48.6, 24.2‐71.8
Last LVPWT NH assessment,^d^ mm			
N (%)^b^	41 (75.9)	25 (56.8)	66 (67.3)
Mean (SD)	10.2 (3.0)	9.3 (2.8)	9.9 (2.9)
Median, range	10.0, 5.0‐17.0	9.0, 6.0‐18.0	9.0, 5.0‐18.0
LVPWT categories, n (%)^b^			
<10 mm	19 (46.3)	19 (76.0)	38 (57.6)
≥10 mm	22 (53.7)	6 (24.0)	28 (42.4)
Age at last LVPWT NH assessment,^d^ years			
N (%)^b^	41 (75.9)	25 (56.8)	66 (67.3)
Mean (SD)	40.7 (15.5)	36.8 (17.6)	39.2 (16.3)
Median, range	44.8, 14.7‐66.1	31.9, 5.7‐76.8	43.1, 5.7‐76.8

Abbreviations: ERT, enzyme replacement therapy; IVST, interventricular septum thickness; LVPWT, left ventricular posterior wall thickness; NH, natural history.

a Percentage based on the total number of patients in each group.

b Percentage based on the number of patients with available IVST/LVPWT data in each group.

c *P* = .93.

d Last NH assessment = most recent value within a 2‐year window before start of ERT (“ERT‐recipients”) or before the last follow‐up date (“ERT‐naïve”).

e *P* = .40.

The median ages at first abnormal LVPWT value (≥10.0 mm^11^) were similar (49.5 [n = 26, 48.1%] vs 46.0 years [n = 13, 29.5%]; *P* = 0.40). Last assessments of LVPWT were performed at older median age in ERT‐recipients (44.8 [n = 41] vs 31.9 years [n = 25]). The median LVPWT values were similar for both groups (10.0 vs 9.0 mm). The percentages of patients with a last assessment of ≥10.0 mm were 53.7% and 24.0%, respectively.

Tables S2 and S3 present the results of cross‐sectional analysis of IVST and LVPWT data using age groups. Although the numbers of patients and assessments varied across the age groups, particularly median IVST values increased with advancing age in ERT‐recipients reaching a moderately abnormal median value of 13.2 mm for 16 females aged 50 to <60 years. However, the results warrant careful interpretations due to limited numbers in each age group.

### 3.4 Renal function measures

eGFR data were available for 56 ERT‐recipients (60.2%) and 51 ERT‐naïve females (67.1%) (Table 4). The median ages at first eGFR <60 mL/min/1.73 m^2^, indicating chronic kidney disease, were similar for both groups (54.8 [n = 8, 14.3%] vs 47.4 years [n = 7, 13.7%]; *P* = 0.40). At the last assessment performed at median ages of 46.8 (n = 42) and 32.1 years (n = 30), respectively, median eGFR values for both groups were similar (99.9 vs 103.8 mL/min/1.73 m^2^), as were the distributions over eGFR categories.

**Table 4:** eGFR assessments during natural history follow‐up in the Fabry Registry

	ERT‐recipients (n = 93)	ERT‐naïve (n = 76)	Overall (n = 169)
Patients with ever eGFR, n (%)^a^	56 (60.2)	51 (67.1)	107 (63.3)
Age at first eGFR <60 mL/min/1.73 m^2^, years			
N (%)^b^	8 (14.3)	7 (13.7)	15 (14.0)
Mean (SD)	56.0 (8.6)^c^	49.8 (11.1)^c^	53.1 (10.0)
Median, range	54.8, 42.2‐69.4	47.4, 32.8‐67.8	54.8, 32.8‐69.4
Last eGFR NH assessment,^d^ mL/min/1.73 m^2^			
N (%)^b^	42 (75)	30 (58.8)	72 (67.3)
Mean (SD)	92.1 (27.7)	94.1 (34.6)	92.9 (30.5)
Median, range	99.9, 3.8‐135.9	103.8, 5.3‐135.5	102.8, 3.8–135.9
eGFR categories, n (%)^b^			
≥60 mL/min/1.73 m^2^	34 (81.0)	26 (86.7)	60 (83.3)
<60 mL/min/1.73 m^2^	8 (19.0)	4 (13.3)	12 (16.7)
Age at last eGFR NH assessment,^d^ years			
N (%)^b^	42 (75)	30 (58.8)	72 (67.3)
Mean (SD)	45.3 (12.7)	37.3 (14.3)	42.0 (13.9)
Median, range	46.8, 20.9‐69.4	32.1, 19.2‐69.2	44.2, 19.2‐69.4

Abbreviations: eGFR, estimated glomerular filtration rate; ERT, enzyme replacement therapy; NH, natural history.

a Percentage based on the total number of patients in each group.

b Percentage based on the number of patients with available eGFR data in each group.

c *P* = .40.

d Last NH assessment = most recent value within a 2‐year window before start of ERT (“ERT‐recipients”) or before the last follow‐up date (“ERT‐naïve”).

The eGFR assessment across age groups showed that the median eGFR values generally decreased with advancing age, but remained above 60 mL/min/1.73 m^2^ for both ERT‐recipients and ERT‐naïve female patients (Table S4).

The numbers of females with available recent UACR and UPCR data were low (Table S5). Moreover, the wide range of UACR values reflects the impact of patient outliers.

### 3.5 Severe clinical events

Relatively few females ever reported a severe event during natural history follow‐up. The percentages for the specific types of events were (ERT‐recipients and ERT‐naïve, respectively): cardiovascular events: 4.3%, 2.6% (significant cardiac procedures in 3.2%, 1.3%); cerebrovascular events: 10.8%, 3.9% (stroke: 3.2%, 1.3%; TIA: 7.5%, 3.9%); dialysis/kidney transplantation: 1.1%, 5.3% (Table S6).

## 4 DISCUSSION

Our Fabry Registry study provides demographics and natural history clinical characteristics of Latin American female patients with Fabry disease with a breakdown of patients who did eventually receive ERT (ERT‐recipients) and those who did not (ERT‐naïve). We were particularly interested in comparing these two groups with regard to clinical presentation, timing of diagnosis, and the natural course of Fabry disease.

Females in both groups often experienced a neurologic symptom as their first Fabry disease manifestation (mostly peripheral pain) in the second decade of life. They carried a high symptomatic burden during (early) adulthood, with numerically higher rates of all Fabry‐related symptoms in ERT‐recipients at older ages compared with ERT‐naïve patients, consistent with the progressive nature of the disease. Yet, female patients were at risk of being diagnosed late; ERT‐recipients were diagnosed at a significantly older age (median 39.2 vs 24.4 years). The long diagnostic delays (median 13.1 vs 7.8 years), despite a predominance of females with a family history, suggests that early case ascertainment as a consequence of family screening^12^ has been the exception rather than the rule. It follows that most female patients are being referred to a specialized center with Fabry disease expertise late and eventually diagnosed on the basis of a history of considerable symptomatic burden. Other Fabry Registry studies of 1077 females from all geographic regions^4^ and of 167 from 8 Latin American countries (also including Mexico, Peru, Uruguay, and Venezuela)^13^ reported median ages at diagnosis of around 30 years and a diagnostic delay of 11.4 years. Increased diagnostic vigilance is needed in order to avoid misdiagnoses due to the often unspecific nature of early Fabry symptoms^14^, ^15^, ^16^ and to counter the significant burden of disease.^8^, ^17^, ^18^, ^19^, ^20^, ^21^, ^22^, ^23^, ^24^ In females, the presence of specific Fabry mutations confirms the diagnosis and the type of mutation may be of predictive value regarding the course of the disease.^7^ In our cohort, 46.7% of the females had a *GLA* mutation associated with a classic phenotype.

We found higher percentages of ERT‐recipients reporting Fabry clinical symptoms during natural history follow‐up, although fewer females had data available, compared with ERT‐naïve patients. Moreover, ERT‐recipients were characterized by cardiovascular involvement, with more patients in the IVST ≥ 10.0 mm category (62.5% vs 42.3%) and the LVPWT ≥ 10.0 mm category (53.7% vs 24.0%) at last natural history assessment, as compared with ERT‐naïve females. This could be because they were diagnosed at an older age (39.2 vs 24.4 years) and therefore had progressed to a more advanced stage of Fabry disease. It is noteworthy that functional renal assessments (medians) were normal and similar for both groups. It should be noted that, as in male Fabry patients, female patients may develop myocardial fibrosis before the development of left ventricular hypertrophy (LVH), suggesting that LVH may not be the only driver of cardiac disease burden in female patients.^25^, ^26^

More ERT‐recipients experienced a cerebrovascular event (10.8% vs 3.9%) and, overall, renal events were the least common type of severe event. Compared with other Fabry Registry studies that used the same severe event definitions,^4^, ^13^ disparate results were only found for cardiovascular complications, the most common cause of death in female patients.^27^, ^28^ These events occurred at a lower rate (4.3% vs 2.6%) as compared with the previous studies (13.9%^4^ and 20.5%^13^). Differences in the populations of female patients and underreporting may have contributed to the disparate findings.

It is encouraging that for ERT‐recipients, there was only a few years delay between diagnosis (age 39.2 years) and initiation of treatment (age 43.2 years), but it is worrisome that there was a large proportion of females reporting Fabry symptoms who never received ERT. For example, around 70% of patients in the ERT‐naïve group reported peripheral pain and abnormal sweating, 66% and 54% intolerance to high and low temperatures, respectively, and around 24% pain crises and GI symptoms. Reluctance to treat symptomatic females has been previously reported in other countries.^29^ One of the barriers could be access to disease‐specific treatment within Latin America.^30^ The most recently published guidelines provide clear recommendations for starting disease‐specific treatment in females with Fabry disease.^8^, ^9^ Briefly, signs or symptoms suggesting major organ involvement (including but not limited to neuropathic pain or pain crises, recurrent diarrhea, impaired sweating) warrant initiation of treatment in females with a classic phenotype. Asymptomatic females with a classic phenotype should be considered for treatment if there is laboratory, histological, or imaging evidence of injury to the kidney, heart, or the central nervous system. Periodic thorough monitoring is a prerequisite for detecting early signs of disease progression.

There are several limitations to our analysis. Fabry Registry data are observational and submitted voluntarily, and there may be a selection bias reporting data on more severely affected patients. Limited data were available for several important demographics and clinical characteristics including the ethnicity and age at first Fabry symptom and, for ERT‐recipients, Fabry symptoms reported during natural history follow‐up. Results of cardiac and eGFR natural history assessments were available for approximately 60% of the patients and very limited numbers of patients had repeated measurements for longitudinal analysis. Data collected prior to referral to a specialized center may not have been made available for entry in the Fabry Registry, and not all data may have been transmitted for entry if patients are undergoing (part of the) monitoring outside these centers. Patient reports to the physician and subsequently to the Fabry Registry may have been limited by patient recall. Incomplete data sets for several critical clinical parameters including left ventricular mass index, residual α‐Gal activity, plasma GL‐3, and lyso‐GL‐3 precluded meaningful analysis. Cardiac fibrosis and X‐chromosome inactivation pattern data were not available for analysis. We were unable to meaningfully analyze changes in symptom reports and intensity and frequency, and progression of organ involvement over time in individual patients given the paucity of data, or determine which patient‐specific clinical considerations led, or could have led, to timely initiation of disease‐specific treatment according to published recommendations. The past decades have witnessed improvements in awareness and understanding of Fabry disease, as well as in diagnostic practices. We have not analyzed changes in diagnostic delays and timing of initiation of ERT over time. Finally, Fabry disease has a remarkable genotypic variability and many *GLA* variants have unclassified phenotypes. To minimize the impact of phenotype heterogeneity on the analyses, we restricted the main analysis population to females with *GLA* variants associated with “classic” Fabry disease or variants that had been reported to the Fabry Registry but had not been entered or classified in the fabry-database.org database. This inclusion criterion was supported by a comparison of the demographics, baseline clinical characteristics and last assessments of the groups of female patients having these variants showing that both groups had similar demographic and clinical features (Table S7).

In conclusion, our study confirms that female patients from Latin America do experience symptoms of Fabry disease—predominantly peripheral pain evident from early adolescence—and experience notable delays in diagnosis. Many female patients carry a high symptomatic burden but may not receive sufficiently early disease‐specific treatment. ERT was prescribed late in female patients with older age at diagnosis and advanced disease. There remained many female patients who had been diagnosed at a younger age, had substantial manifestations of Fabry disease, but did not receive disease‐specific treatment.

An increase in awareness of the natural history of Fabry disease in female patients and the consequences of late initiation of treatment, and the encouragement of frequent comprehensive monitoring and family pedigree analysis can help identify female patients at an earlier age. Once the patients are diagnosed, timely initiation of treatment could be enabled by researching and understanding the non‐clinical prompts and barriers to starting disease‐specific treatment in female patients in Latin America who suffer from this progressive disease.

## CONFLICT OF INTERESTS

A.M.M. is a Fabry Registry Board member and received research funds, travel support, and speaking fees from Sanofi Genzyme, Alexion, and Biomarin. G.C. has consulting arrangements with and received speaking fees from Sanofi Genzyme. He received travel support from Sanofi Genzyme and Takeda. F.M. is a Fabry Registry Board member and received honoraria for lectures and board meetings from Sanofi Genzyme and speaking fees from Takeda and Merck Serono. F.S‐O. is a Fabry Registry Board member and received research funds, travel support, and speaking fees from Sanofi Genzyme. R.A.V. is a Fabry Registry Board member and received travel support and speaking fees from Sanofi Genzyme. C.V. is a Fabry Registry Board member and received honoraria for lectures and board meetings from Sanofi Genzyme and speaking fees from Takeda. M.Y. is an employee of Sanofi Genzyme. J.M.P. received honoraria for presentations and board meetings from Sanofi Genzyme, Takeda, and Amicus Therapeutics.

## AUTHOR CONTRIBUTIONS

A.M.M., G.C., F.M., F.S‐O., C.V., and J.M.P. contributed to the data acquisition, data analysis/interpretation, drafting the manuscript, and critically revising it. R.A.V. contributed to the data interpretation, drafting the manuscript, and critically revising it. M.Y. contributed to the data analysis/interpretation, drafting the manuscript, and critically revising it.

## GUARANTOR

A.M.M. accepts full responsibility for the work and/or the conduct of the study, had access to the data, and controlled the decision to publish.

## INFORMED CONSENT

Patients' informed written consent to submit their health information to the Fabry Registry and to disclose this information in further analyses is obtained by each independent site.

## ETHICS COMMITTEE APPROVAL

The Fabry Registry protocol, informed consent form, and any locally required authorization documents are reviewed and approved by the local fully constituted Institutional Review Board or Independent Ethics Committee unless the site provides the Registry with documentation that approval is not required or has been waived.

## Supporting information


**Table S1** First manifestation of Fabry disease present at onset by organ system
**Table S2** IVST assessments during natural history follow‐up in the Fabry Registry by age groups
**Table S3** LVPWT assessments during natural history follow‐up in the Fabry Registry by age groups
**Table S4** eGFR assessments during natural history follow‐up in the Fabry Registry by age groups
**Table S5** UACR and UPCR assessments during natural history follow‐up in the Fabry Registry
**Table S6** Severe clinical events occurring during natural history follow‐up in the Fabry Registry
**Table S7** Demographics and clinical characteristics during natural history follow‐up in the Fabry Registry by phenotype

## Data Availability

Qualified researchers may request access to patient level data and related study documents including the clinical study report, study protocol with any amendments, blank case report form, statistical analysis plan, and dataset specifications. Patient level data will be anonymized, and study documents will be redacted to protect the privacy of trial participants. Further details on Sanofi's data sharing criteria, eligible studies, and process for requesting access can be found at: https://www.clinicalstudydatarequest.com.
