# Supplementary material for: The clinical profiles of female patients with Fabry disease in Latin America: A Fabry Registry analysis of natural history data from 169 patients based on enzyme replacement therapy status
Source: JIMD Rep. 2019 Aug 5;49(1):107–17. doi: 10.1002/jmd2.12071 (PMC6718114; doi:10.1002/jmd2.12071)
Supplement: Supplementary file 1 — Table S1 First manifestation of Fabry disease present at onset by organ system Table S2 IVST assessments during natural history follow‐up in the Fabry Registry by age groups Table S3 LVPWT assessments during natural history follow‐up in the Fabry Registry by age groups Table S4 eGFR assessments during natural history follow‐up in the Fabry Registry by age groups Table S5 UACR and UPCR assessments during natural history follow‐up in the Fabry Registry Table S6 Severe clinical events occurring during natural history follow‐up in the Fabry Registry Table S7 Demographics and clinical characteristics during natural history follow‐up in the Fabry Registry by phenotype [file JMD2-49-107-s001.docx]

**Supplementary Table S1** First manifestation of Fabry disease present at onset by organ system

|  | **ERT-recipients**  **(n=93)** | **ERT-naïve (n=76)** | **Overall (n=169)** |
| --- | --- | --- | --- |
| First manifestation of FD, n (%) | 56 (60.2) | 38 (50.0) | 94 (55.6) |
| Neurologic | 45 (80.4) | 32 (84.2) | 77 (81.9) |
| Peripheral pain | 37 (66.1) | 24 (63.2) | 61 (64.9) |
| Gastrointestinal | 6 (10.7) | 3 (7.9) | 9 (9.6) |
| Skin | 3 (5.4) | 4 (10.5) | 7 (7.4) |
| Ophthalmologic | 5 (8.9) | 0 | 5 (5.3) |
| Cardiac | 3 (5.4) | 3 (7.9) | 6 (6.4) |
| Renal | 3 (5.4) | 3 (7.9) | 6 (6.4) |
| Cerebrovascular | 3 (5.4) | 1 (2.6) | 4 (4.3) |
| Other | 2 (3.6) | 1 (2.6) | 3 (3.2) |

Percentages are based on the total number of patients in each group

*ERT*, enzyme replacement therapy; *FD*, Fabry disease

**Supplementary Table S2** IVST assessments during natural history follow-up in the Fabry Registry by age groups

|  | **Age at IVST assessment(s)** | | | | |
| --- | --- | --- | --- | --- | --- |
|  | **18 - <30 years** | **30 - <40 years** | **40 - <50 years** | **50 - <60 years** | **≥60 years** |
| **ERT-recipients: IVST, mm** | | | | | |
| Patients, n^a^ | 7 | 5 | 20 | 16 | 3 |
| Assessments, n^b^ | 11 | 6 | 31 | 24 | 5 |
| Mean (SD) | 8.3 (1.5) | 10.0 (2.8) | 12.0 (3.4) | 13.5 (4.0) | 14.8 (5.5) |
| Median, range | 8.0, 7.0–12.0 | 10.0, 6.0–13.0 | 11.0, 7.0–19.0 | 13.2, 8.0–23.0 | 14.0, 9.0–21.0 |
| **ERT-naïve: IVST, mm** | | | | | |
| Patients, n^a^ | 15 | 4 | 8 | 10 | 1 |
| Assessments, n^b^ | 20 | 9 | 19 | 25 | 4 |
| Mean (SD) | 7.6 (1.3) | 8.7 (1.2) | 10.9 (2.4) | 12.6 (4.6) | 16.0 (3.6) |
| Median, range | 7.5, 6.0–11.0 | 9.0, 7.0–10.0 | 10.0, 7.3–17.0 | 10.5, 7.0–23.0 | 15.5, 13.0–20.0 |
| **Overall: IVST, mm** | | | | | |
| Patients, n^a^ | 22 | 9 | 28 | 26 | 4 |
| Assessments, n^b^ | 31 | 15 | 50 | 49 | 9 |
| Mean (SD) | 7.8 (1.4) | 9.2 (2.0) | 11.6 (3.1) | 13.0 (4.3) | 15.3 (4.5) |
| Median, range | 8.0, 6.0–12.0 | 9.0, 6.0–13.0 | 11.0, 7.0–19.0 | 11.0, 7.0–23.0 | 14.0, 9.0–21.0 |

*ERT*, enzyme replacement therapy; *IVST*, interventricular septum thickness; *SD*, standard deviation

^a^ Number of patients included in each specific age group of assessment. A patient may be in several groups if she has multiple records assessed at different ages

^b^ Number of assessments included in each specific age group of assessment. A patient may have multiple records in each age group

**Supplementary Table S3** LVPWT assessments during natural history follow-up in the Fabry Registry by age groups

|  | **Age at LVPWT assessment(s)** | | | | |
| --- | --- | --- | --- | --- | --- |
|  | **18 - <30 years** | **30 - <40 years** | **40 - <50 years** | **50 - <60 years** | **≥60 years** |
| **ERT-recipients: LVPWT, mm** | | | | | |
| Patients, n^a^ | 9 | 5 | 20 | 15 | 3 |
| Assessments, n^b^ | 13 | 5 | 31 | 23 | 5 |
| Mean (SD) | 7.7 (1.5) | 9.0 (3.0) | 10.9 (2.8) | 11.4 (2.6) | 12.2 (1.6) |
| Median, range | 7.4, 5.0–11.0 | 8.0, 6.0–14.0 | 10.0, 7.0–17.0 | 11.0, 7.0–16.0 | 13.0, 10.0–14.0 |
| **ERT-naïve: LVPWT, mm** | | | | | |
| Patients, n^a^ | 14 | 5 | 8 | 10 | 1 |
| Assessments, n^b^ | 19 | 10 | 18 | 23 | 4 |
| Mean (SD) | 7.5 (1.2) | 9.9 (4.4) | 10.4 (1.9) | 11.1 (3.3) | 13.0 (2.2) |
| Median, range | 8.0, 5.0–9.0 | 9.0, 7.0–22.0 | 10.1, 8.0–15.0 | 9.6, 8.0–18.0 | 12.5, 11.0–16.0 |
| **Overall: LVPWT, mm** | | | | | |
| Patients, n^a^ | 23 | 10 | 28 | 25 | 4 |
| Assessments, n^b^ | 32 | 15 | 49 | 46 | 9 |
| Mean (SD) | 7.6 (1.3) | 9.6 (3.9) | 10.7 (2.5) | 11.2 (2.9) | 12.6 (1.8) |
| Median, range | 7.7, 5.0–11.0 | 9.0, 6.0–22.0 | 10.0, 7.0–17.0 | 10.5, 7.0–18.0 | 13.0, 10.0–16.0 |

*ERT*, enzyme replacement therapy; *LVPWT*, left ventricular posterior wall thickness; *SD*, standard deviation

^a^ Number of patients included in each specific age group of assessment. A patient may be in several groups if she has multiple records assessed at different ages

^b^ Number of assessments included in each specific age group of assessment. A patient may have multiple records in each age group

**Supplementary Table S4** eGFR assessments during natural history follow-up in the Fabry Registry by age groups

|  | **Age at eGFR assessment(s)** | | | | |
| --- | --- | --- | --- | --- | --- |
|  | **18 - <30 years** | **30 - <40 years** | **40 - <50 years** | **50 - <60 years** | **≥60 years** |
| **ERT-recipients: eGFR, ml/min/1.73m^2^** | | | | | |
| Patients, n^a^ | 9 | 5 | 17 | 15 | 5 |
| Assessments, n^b^ | 13 | 10 | 33 | 30 | 8 |
| Mean (SD) | 117.1 (18.1) | 108.0 (20.9) | 100.7 (22.1) | 81.6 (23.3) | 79.0 (18.2) |
| Median, range | 121.2, 89.3–142.0 | 115.4, 73.0–132.8 | 105.2, 3.8–126.8 | 80.2, 43.1–119.8 | 88.6, 49.0–94.5 |
| **ERT-naïve: eGFR, ml/min/1.73m^2^** | | | | | |
| Patients, n^a^ | 17 | 10 | 6 | 9 | 3 |
| Assessments, n^b^ | 40 | 22 | 15 | 25 | 9 |
| Mean (SD) | 115.2 (15.7) | 108.3 (24.5) | 74.3 (22.0) | 60.9 (36.2) | 73.3 (15.1) |
| Median, range | 117.9, 84.4–137.1 | 115.5, 22.4–134.2 | 80.7, 29.8–103.5 | 76.1, 4.7–101.1 | 77.6, 42.2–93.8 |
| **Overall: eGFR, ml/min/1.73m^2^** | | | | | |
| Patients, n^a^ | 26 | 15 | 23 | 24 | 8 |
| Assessments, n^b^ | 53 | 32 | 48 | 55 | 17 |
| Mean (SD) | 115.7 (16.2) | 108.2 (23.1) | 92.5 (25.1) | 72.2 (31.4) | 76.0 (16.3) |
| Median, range | 117.9, 84.4–142.0 | 115.5, 22.4–134.2 | 98.5, 3.8–126.8 | 77.6, 4.7–119.8 | 79.1, 42.2–94.5 |

*eGFR*, estimated glomerular filtration rate; *ERT*, enzyme replacement therapy; *SD*, standard deviation

^a^ Number of patients included in each specific age group of assessment. A patient may be in several groups if she has multiple records assessed at different ages

^b^ Number of assessments included in each specific age group of assessment. A patient may have multiple records in each age group

**Supplementary Table S5** UACR and UPCR assessments during natural history follow-up in the Fabry Registry

|  | **ERT-recipients**  **(n=93)** | **ERT-naïve**  **(n=76)** | **Overall**  **(n=169)** |
| --- | --- | --- | --- |
| Last spot/24hr UACR NH assessment^a^, mg/g  N (%)^b^ | 19 (20.4) | 12 (15.8) | 31 (18.3) |
| Mean (SD)  Median, range | 250.9 (347.0)  114.3, 1.5–1368 | 114.8 (328.9)  16.4, 2.0–1158 | 198.3 (341.2)  43.7, 1.5–1368 |
| UACR categories, n (%)^c^ |  |  |  |
| <30 mg/g | 5 (26.3) | 8 (66.7) | 13 (41.9) |
| ≥30 mg/g | 14 (73.7) | 4 (33.3) | 18 (58.1) |
| Age at last UACR NH assessment^a^, years  Mean (SD)  Median, range | 43.9 (16.4)  49.8, 14.7–67.6 | 26.8 (14.4)  23.7, 9.6–55.4 | 37.3 (17.6)  33.3, 9.6–67.6 |
| Last spot/24hr UPCR NH assessment^a^, g/g |  |  |  |
| N (%)^b^ | 26 (28.0) | 18 (23.7) | 44 (26.0) |
| Mean (SD)  Median, range | 0.2 (0.3)  0.1, 0–1.4 | 0.3 (0.6)  0, 0–2.7 | 0.2 (0.5)  0.1, 0–2.7 |
| UPCR categories, n (%)^c^ |  |  |  |
| <0.5 g/g | 25 (96.2) | 16 (88.9) | 41 (93.2) |
| ≥0.5 g/g | 1 (3.8) | 2 (11.1) | 3 (6.8) |
| Age at last UPCR NH assessment^a^, years  Mean (SD)  Median, range | 41.4 (15.2)  45.7, 14.7–67.6 | 26.2 (13.4)  23.7, 9.6–55.4 | 35.2 (16.2)  33.0, 9.6–67.6 |

*ERT*, enzyme replacement therapy; *NH*, natural history; *SD*, standard deviation; *UACR*, urine albumin-to-creatinine ratio; *UPCR*, urine protein-to-creatinine ratio

^a^ Last NH assessment = most recent value within a 2-year window before start of ERT (“ERT-recipients”) or before the last follow-up date (“ERT-naïve”)

^b^ Percentage based on the total number of patients in each group

^c^ Percentage based on the number of patients with available UACR/UPCR data in each group

**Supplementary Table S6** Severe clinical events occurring during natural history follow-up in the Fabry Registry

| **Severe clinical events,**  **age at first report** | **ERT-recipients**  **(n=93)** | **ERT-naïve**  **(n=76)** | ***p*-value** | **Overall**  **(n=169)** |
| --- | --- | --- | --- | --- |
| Any cardiovascular event, n (%)^a^ | 4 (4.3) | 2 (2.6) |  | 6 (3.6) |
| Significant cardiac procedure  n (%)  Mean (SD)  Median, range  Atrial fibrillation  n (%)  Mean (SD)  Median, range  Ventricular tachycardia  n (%)  Mean (SD)  Median, range  Angina pectoris  n (%)  Mean (SD)  Median, range | 3 (3.2)  57.8 (13.0)  52.1, 48.6–72.7  –  –  1 (1.1)  42.1 (–)  42.1, 42.1–42.1 | 1 (1.3)  57.0 (–)  57.0, 57.0–57.0  1 (1.3)  56.1 (–)  56.1, 56.1–56.1  1 (1.3)  49.3 (–)  49.3, 49.3–49.3  – | 1.00  –  –  – | 4 (2.4)  57.6 (10.7)  54.5, 48.6–72.7  1 (0.6)  56.1 (–)  56.1, 56.1–56.1  1 (0.6)  49.3 (–)  49.3, 49.3–49.3  1 (0.6)  42.1 (–)  42.1, 42.1–42.1 |
| Chronic dialysis/kidney transplant  n (%)  Mean (SD)  Median, range | 1 (1.1)  28.2 (–)  28.2, 28.2–28.2 | 4 (5.3)  41.4 (6.9)  39.6, 35.4–50.9 | 0.35 | 5 (3.0)  38.7 (8.4)  37.4, 28.2–50.9 |
| Any cerebrovascular event, n (%) | 10 (10.8) | 3 (3.9) |  | 13 (7.7) |
| Stroke  n (%)  Mean (SD)  Median, range  TIA  n (%)  Mean (SD)  Median, range | 3 (3.2)  44.4 (9.9)  42.3, 35.9–55.2  7 (7.5)  45.0 (11.9)  49.6, 27.9–59.5 | 1 (1.3)  25.5 (–)  25.5, 25.5–25.5  3 (3.9)  30.9 (12.4)  24.6, 22.9–45.2 | 0.44  0.15 | 4 (2.4)  39.7 (12.4)  39.1, 25.5–55.2  10 (5.9)  40.7 (13.2)  44.9, 22.9–59.5 |

All ages in years. Percentages are based on the total number of patients in each group

*ERT*, enzyme replacement therapy; *SD*, standard deviation; *TIA*, transient ischemic attack

^a^ There were no reports of congestive heart failure, myocardial infarction, or cardiac syncope

**Supplementary Table S7** Demographics and clinical characteristics during natural history follow-up in the Fabry Registry by phenotype

|  | **Classic phenotype** | **Unclassified**  **phenotype** |
| --- | --- | --- |
| Total number of patients, n | 79 | 90 |
| Age at FD diagnosis, years, n (%) | 77 (97.5) | 89 (98.9) |
| Mean (SD) | 33.9 (18.2) | 30.8 (17.3) |
| Median, range | 34.2, 0.4–72.0 | 28.7, 0.4–71.8 |
| Age at FD symptom (any) onset, years, n (%) | 44 (55.7) | 50 (55.6) |
| Mean (SD) | 19.3 (14.6) | 15.2 (12.6) |
| Median, range | 15.8, 2.6–67.9 | 11.7, 0–48.9 |
| Age at first NH assessment, years, n (%) | 72 (91.1) | 85 (94.4) |
| Mean (SD) | 21.4 (16.0) | 23.5 (17.3) |
| Median, range | 17.2, 0.4–67.6 | 19.8, 0–65.9 |
| Age at last NH assessment, years, n (%) | 72 (91.1) | 85 (94.4) |
| Mean (SD) | 37.4 (18.4) | 35.6 (16.3) |
| Median, range | 35.9, 1.5–73.7 | 33.3, 1.1–76.8 |
| ACEi/ARBs use during NH, n (%) | 79 (100) | 90 (100) |
| Ever use, n (%) | 2 (2.5) | 8 (8.9) |
| Never use, n (%) | 77 (97.5) | 82 (91.1) |
| Last IVST NH assessment, mm, n (%)^a^ | 28 (35.4) | 59 (65.6) |
| Mean (SD) | 9.7 (2.3) | 11.0 (4.0) |
| Median, range | 9.0, 7.0–17.0 | 10.0, 6.0–23.0 |
| Last LVPWT NH assessment, mm, n (%)^a^ | 28 (35.4) | 61 (67.8) |
| Mean (SD) | 9.2 (2.0) | 10.0 (3.4) |
| Median, range | 9.0, 6.0–14.9 | 9.0, 5.0–22.0 |
| Last eGFR NH assessment, ml/min/1.73m^2^, n (%)^a^ | 33 (41.8) | 51 (56.7) |
| Mean (SD) | 92.5 (24.4) | 94.3 (32.1) |
| Median, range | 94.5, 32.7–130.4 | 103.3, 3.8–135.9 |
| Age at first ERT, years, n (%) | 38 (48.1) | 36 (40.0) |
| Mean (SD) | 43.3 (16.9) | 35.4 (15.1) |
| Median, range | 42.3, 12.9–73.7 | 31.9, 3.8–60.6 |

*ACEi,* angiotensin-converting enzyme inhibitor; *ARB*, angiotensin receptor blocker; *eGFR*, estimated glomerular filtration rate; *ERT*, enzyme replacement therapy; *FD,* Fabry disease; *IVST,* interventricular septum thickness; *LVPWT*, left ventricular posterior wall thickness; NH, natural history; *SD*, standard deviation

^a^ Last NH assessment = most recent value within a 2-year window before start of ERT (“ERT-recipients”) or before the last follow-up date (“ERT-naïve”)
